# Exosomal miR-361-3p promotes the viability of breast cancer cells by targeting ETV7 and BATF2 to upregulate the PAI-1/ERK pathway

**DOI:** 10.1186/s12967-024-04914-4

**Published:** 2024-01-28

**Authors:** Yao Li, Lei Fan, An Yan, Xiaotian Ren, Yanyang Zhao, Bin Hua

**Affiliations:** 1 Breast center, Department of Thyroid-Breast-Hernia Surgery, Department of General Surgery, Beijing Hospital, National Center of Gerontology, Institute of Geriatric Medicine, Chinese Academy of Medical Sciences, Beijing, People’s Republic of China; 2 The Key Laboratory of Geriatrics, Beijing Institute of Geriatrics, Institute of Geriatric Medicine, Chinese Academy of Medical Sciences, Beijing Hospital/National Center of Gerontology of National Health Commission, Beijing, People’s Republic of China

**Keywords:** Breast cancer, Malignant progression, Exosomal miR-361-3p, Migration, Proliferation

## Abstract

**Background:**

Malignant progression is the major cause of poor prognosis in breast cancer (BC) patients. Plasma exosomal miRNAs have been reported to be involved in tumor progression, but their roles in BC remain unclear.

**Methods:**

We performed plasma exosomal miRNA sequencing on 45 individuals, including healthy controls and nonmetastatic and metastatic BC patients. We examined the correlation between miRNA expression in tumor tissues and plasma exosomes in BC patients by qRT‒PCR. The effects of exosomal miR-361-3p on BC cells were determined by CellTiter-Glo, migration and wound healing assays. The target genes of miR-361-3p and downstream pathways were explored by dual-luciferase reporter assay, RNA knockdown, rescue experiments, and western blotting. We utilized murine xenograft model to further assess the impact of plasma exosomal miR-361-3p on the malignant progression of BC.

**Results:**

We found that the expression level of plasma exosomal miR-361-3p gradually increased with malignant progression in BC patients, and the expression of miR-361-3p in plasma exosomes and BC tissues was positively correlated. Consistently, exosomal miR-361-3p enhanced the migration and proliferation of two BC cell lines, MDA-MB-231 and SK-BR-3. Furthermore, our data showed that miR-361-3p inhibited two novel target genes, *ETV7* and *BATF2*, to activate the PAI-1/ERK pathway, leading to increased BC cell viability. Finally, the consistency of the in vivo experimental results supported that elevated plasma exosomal miR-361-3p promote the malignant progression of BC.

**Conclusions:**

We found for the first time that plasma exosomal miR-361-3p was associated with malignant progression in BC patients. Mechanistically, exosomal miR-361-3p can enhance the migration and proliferation of BC cells by targeting the ETV7 and BATF2/PAI-1/ERK pathways. Our data suggest that plasma exosomal miR-361-3p has the potential to serve as a biomarker for predicting malignant progression in BC patients.

**Supplementary Information:**

The online version contains supplementary material available at 10.1186/s12967-024-04914-4.

## Background

Breast cancer (BC) is the most common malignant tumor among females, with over 2.3 million cases globally in 2023. Despite significant advances in diagnostic and therapeutic techniques for BC, which have somewhat improved the prognosis for patients, BC still accounts for 15% of global tumor-related deaths in adult women [1–4]. Malignant progression is one of the major causes of death in BC patients, but its early prediction and control remain serious challenges [5–8].

Exosomes are tiny vesicles ubiquitously present in body fluids, tissues and cells. Due to their stable structure, exosomes protect their cargoes from degradation. In recent years, many studies have demonstrated that plasma exosomes can serve as biomarkers to assess tumor occurrence, progression and prognosis. As one of the most abundant nucleic acid species in exosomes, miRNAs have been extensively investigated [9–12]. For instance, Aiko Sueta et al., by comparing the serum exosomal miRNA profiles in 16 recurrent and 16 non-recurrent BC patients, identified 11 differentially expressed genes significantly associated with tumor recurrence, further proposing miR-340-5p, miR-17-5p, miR-130a-3p, and miR-93-5p as potential biomarkers for predicting BC [13]. Ines Stevic et al. conducted a microarray analysis of 45 miRNAs in plasma exosomes from BC patients before and after neoadjuvant therapy in the GeparSixto trial, finding that miR-155 and miR-301 could be potential markers for predicting pathological complete response [14]. Hannafon Bethany N. et al. pointed out that, compared to healthy women, plasma exosomal miR-21 and miR-1246 are significantly elevated in BC patients and can distinguish them from healthy control [15]. Additionally, emerging evidence suggests that exosomal miRNAs play an important role in intercellular communication. For example, one study indicated that exosomal miR-181b-5p can induce resistance to doxorubicin in BC cells by regulating *BCLAF1* [16]. Another study reported that macrophage-derived exosomal miR-181a activates the AKT pathway in BC cells [17]. Furthermore, research has shown that exosomal miR-500a-5p from fibroblasts can target *USP28* in BC cells to promote tumor proliferation and metastasis [18]. However, the exact role and mechanisms of exosomal miRNAs in the malignant progression of BC remain unclear.

In our research, we performed plasma exosomal miRNA sequencing (miRNA-seq) and discovered that elevated plasma exosomal miR-361-3p was positively correlated with malignant progression in BC patients. Mechanistically, exosomal miR-361-3p can enhance the viability of BC cells by inhibiting *ETV7* and *BATF2* expression, thereby upregulating the PAI-1/ERK pathway. Together, our results suggest that exosomal miR-361-3p is a potential biomarker for predicting malignant progression in BC patients.

## Materials and methods

### Patient information and sample collection

For this study, we recruited 8 healthy control, 32 nonmetastatic and 5 metastatic BC patients between April 2021 and May 2022 at Beijing Hospital. Blood samples were collected from all participants in 5 mL vacutainer tubes containing EDTA as an anticoagulant. After centrifugation of the blood samples at 1500 × g for 10 min at 4 °C, followed by 3000 × g for 15 min at 4 °C, 2 ml of plasma was aspirated and stored at − 80 °C for subsequent isolation and sequencing of exosomal miRNA. Additionally, 18 BC tissue samples were obtained from the aforementioned BC patients and preserved in liquid nitrogen. Detailed clinical information of the 37 BC patients is presented in Table 1.

**Table 1:** Clinical characteristics of the 37 female breast cancer patients in plasma exosomal miRNA sequencing analysis

Sample ID	Age of diagnosis (yr)	Metastatic/non-metastatic BC	Tumor size (mm)*	Stage of cancer (TNM)^†^	Molecular subtypes^‡^
BC-A192-03	69	Non-metastasis	21	II	Luminal B
BC-A192-04	64	Non-metastasis	42	II	Luminal B
BC-A192-05	68	Non-metastasis	36	II	TNBC
BC-A192-06	66	Non-metastasis	29	II	HER2 +
BC-A192-07	30	Non-metastasis	11	III	HER2 +
BC-A192-08	54	Non-metastasis	41	II	Luminal B
BC-A192-09	39	Non-metastasis	27	II	HER2 +
BC-A192-14	45	Non-metastasis	36	II	TNBC
BC-A192-15	34	Non-metastasis	15	II	HER2 +
BC-A192-16	57	Non-metastasis	24	II	Luminal B
BC-A192-18	54	Non-metastasis	21	II	Luminal A
BC-A192-19	39	Non-metastasis	46	I	Luminal B
BC-A192-20	42	Non-metastasis	23	II	TNBC
BC-A192-21	59	Non-metastasis	26	II	HER2 +
BC-A192-25	66	Non-metastasis	36	II	HER2 +
BC-A192-26	64	Non-metastasis	18	III	Luminal B
BC-A192-27	42	Non-metastasis	27	II	HER2 +
BC-A192-28	58	Non-metastasis	25	II	Luminal B
BC-A192-30	51	Non-metastasis	31	II	HER2 +
BC-A192-31	38	Non-metastasis	39	II	TNBC
BC-A192-50	52	Non-metastasis	11	I	Luminal A
BC-A192-52	65	Non-metastasis	11	I	TNBC
BC-A192-53	46	Non-metastasis	17	I	Luminal A
BC-A192-54	40	Non-metastasis	14	III	Luminal B
BC-A192-60	33	Non-metastasis	6.8	III	TNBC
BC-A192-63	58	Non-metastasis	61	III	Luminal B
BC-A192-69	52	Non-metastasis	09	I	TNBC
BC-A192-71	48	Non-metastasis	31	III	Luminal B
BC-A192-75	38	Non-metastasis	26	III	Luminal A
BC-A192-85	55	Non-metastasis	26	III	HER2 +
BC-A192-95	55	Non-metastasis	31	III	HER2 +
BC-A192-96	58	Non-metastasis	25	III	HER2 +
BC-A192-01	56	Metastasis (Bone)	32	IV	Luminal A
BC-A192-02	64	Metastasis (Bone, Pleural, Liver)	43	IV	TNBC
BC-A192-10	51	Metastasis (Liver, Lung)	24	IV	HER2 +
BC-A192-22	62	Metastasis (Pleural)	13	IV	Luminal B
BC-A192-32	66	Metastasis (Bone, Pleural)	54	IV	HER2 +

^*^The greatest dimension of the tumor measured by ultrasound

^†^Staging was based on the tumor-node-metastasis (TNM) classification of American Joint on Cancer

^‡^The molecular subtype is based on the classification system established by the American Joint Committee on Cancer, which takes into account the expression levels of estrogen receptor (ER), progesterone receptor (PR), human epidermal growth factor receptor 2 (HER2), and Ki-67

All participants provided written informed consent, agreeing to the collection of plasma and tissue samples, as well as the use of their pathological data for the purpose of this study. The study protocol was approved by the Ethics Committee of Beijing Hospital, in accordance with the principles of the Declaration of Helsinki, and written informed consent was obtained from all patients.

### Isolation of plasma exosomes

Plasma exosomes were isolated using size exclusion chromatography. One millilitre of blood plasma, filtered through a 0.8 μm filter, was diluted 1.5 times with PBS and further purified using Exosupur columns (Echobiotech, China). The samples were then eluted with PBS, and 2 mL eluate fractions were collected. Subsequently, these fractions were concentrated down to 200 μL using 100 kDa molecular weight cut-off Amicon Ultra spin filters (Millipore, Germany).

### Identification of plasma exosomes

Exosomes were identified using three methods: nanoparticle tracking analysis (NTA), transmission electron microscopy (TEM), and western blotting (WB). Initially, vesicle suspensions at concentrations of about 1 × 10^8^/mL were analyzed using a ZetaView PMX 110 (Particle Metrix, Meerbusch, Germany) equipped with a 405 nm laser. The size and quantity of the isolated particles were determined. A total of 20 μL of exosomes was analyzed, and particle movement was assessed using NTA software (ZetaView 8.02.28). Subsequently, the exosomes were resuspended in PBS and placed on an electron microscope's copper mesh. After a 10-min incubation at room temperature, 1% uranyl acetate was used for negative staining for 10 min. Observations and images were made with a TEM (H-7650, Hitachi Ltd., Tokyo, Japan). Finally, exosome-enriched supernatant was denatured in 5 × SDS buffer and subjected to WB using TSG101 (sc-13611, Santa Cruz), HSP70 (ab2787, Abcam), CD63 (ab275377, Abcam), and Calnexin (ab92573, Abcam) [19, 20].

### Plasma exosomal miRNA-seq

Each of 45 samples of plasma exosomes yielded approximately 10 ng of RNA, which were extracted using the miRNeasy Kit (Qiagen, Germany). Subsequently, miRNA-seq was performed on an Illumina HiSeq platform. The miRNA-seq data, expressed as Unique Molecular Identifiers (UMIs), were corrected and normalized using the "limma" R package. Differential expression analysis was conducted using the "edgeR" R package. The criteria used for filtering differentially expressed exosomal miRNAs were TPM (transcripts per million) ≥ 30, *P* < 0.05, and |log_2_ fold change (FC)|> 0.5.

### Cell culture, coculture with exosomes, and transfection

The human BC cell lines (MDA-MB-231, SK-BR-3 and T47D) and HEK293T cell line were purchased from the Cell Centre, School of Basic Medicine Peking Union Medical College. Cells were cultured in RPMI-1640 medium with 10% fetal bovine serum in humidified air at 37 °C with 5% CO_2_.

HEK293T cells were used to produce exosomes, which were collected through ultracentrifugation. The exosomes were then loaded with mimic negative control (miR-NC) or miR-361-3p mimic (miR-361-3p) (RiboBio) using the ExoLoadTM kit (Echo Biotech). Subsequently, 200 pmol of miRNA was mixed with the exosomes and the ExoLoadTM reagent, and the mixture was co-incubated at 37°C with 1 × 10^11^ exosomes for 2 h. Following the co-incubation, the mixture was subjected to ultrafiltration using a 100 kDa filter membrane (Millipore) to remove any free miRNA that had not been loaded into the exosomes. Finally, we obtained exosomes loaded with either miR-NC (EXO-miR-NC) or miR-361-3p (EXO-miR-361-3p). During the coculture of BC cells with exosomes, a concentration of 10^5^ particles per cell was used.

MiR-361-3p, miR-NC, miR-361-3p inhibitor, miR-NC inhibitor, siRNA for ETV7 (siETV7), BATF2 (siBATF2), PAI-1 (siPAI-1) and siRNA negative control (siNC) (RiboBio) were transfected into cells by Lipofectamine RNAiMAX (Invitrogen; Thermo Fisher Scientific, Inc.). Human *ETVT* cDNA plasmid (*ETV7* plasmid), *BATF2* cDNA plasmid (*BATF2* plasmid), *PAI-1* cDNA plasmid (*PAI-1* plasmid) and negative control plasmid (control plasmid) were transfected into cells using Lipofectamine 2000 (Invitrogen; Thermo Fisher Scientific, Inc.).

### Evaluation of exosomes uptake via PKH67 fluorescence labeling

Exosomes were labeled using the PKH67 Green Fluorescent Cell Linker Midi Kit (MIDI67-1KT, Sigma-Aldrich, St. Louis, MO) according to the manufacturer's instructions. First, isolated exosomes were mixed with 1 mL of the provided diluent and 4 μL of PKH67 solution and incubated for 4 min. Then, 2 mL of 0.5% bovine serum albumin solution in PBS was added for quenching. The labeled exosomes were subjected to two rounds of ultracentrifugation at 100,000 × g for 70 min each at 4 °C and resuspended in 100 μL of PBS.

To assess exosome uptake, MDA-MB-231 and SK-BR-3 cells were seeded in 24-well plates at a density of 1 × 10^4^ cells per well and incubated for 24 h at 37 °C with 5% CO_2_. Subsequently, 10 μg of PKH67-labeled exosomes was added to each well, and the cells were further incubated for 2 or 12 h at 37 °C with 5% CO_2_. After incubation, the cells were fixed with 4% paraformaldehyde for 20 min and stained with DAPI for nuclear staining. The uptake of exosomes was observed under a fluorescence microscope (KEYENCE LED) at 400 × magnification. The microscopy imaging parameters were set at the initial acquisition and remained constant throughout the acquisition. Green fluorescence represents the PKH67-labeled exosomes.

### Real-time polymerase chain reaction analysis

Total RNA was extracted from BC clinical samples and cells using RNAiso Plus (TaKaRa). A PrimeScript RT reagent Kit (TaKaRa) was used to synthesize miRNA. Quantitative real-time polymerase chain reaction (qRT‒PCR) assays were performed on a 7500 Real-time PCR system (Applied Biosystems) with SYBR Premix Ex Taq (TaKaRa). The level of *U6* expression was used as the internal control for *miR-361-3p*. The relative expression levels of mRNAs were calculated and quantified using the 2^−△△ct^ method. The qRT‒PCR primers were as follows: miR-361-3p stem‒loop RT primer: 5'-GTCGTATCCAGTGCAGGGTCCGAGGTATTCGCACTGGATACGACAAATCAGAATC-3'; miR-361-3p: Forward 5'-GCCGCTCCCCCAGGTGTGATT-3' and reverse 5'-GTGCAGGGTCCGAGGT-3'; U6: Forward 5'-GGCAGGAAGAGGGCCTA-3' and reverse 5'-GTGCAGGGTCCGAGGT-3'. The experiments were conducted in triplicate.

### Western blotting assay

Total proteins were extracted from cells using RIPA buffer (Beyotime Institute of Biotechnology). The proteins were separated by 10% SDS‒PAGE and transferred to a PVDF membrane (EMD Millipore). The membrane was blocked with 5% fat-free milk and incubated with primary monoclonal rabbit anti-ETV7 (1:1000, ab229832, Abcam), mouse anti-ETV7 (1:500, E-1, Santa Cruz), rabbit anti-BATF2 (1:500, 1B11, Santa Cruz), rabbit anti-PAI-1 (1:500, ab182973, Abcam), rabbit anti-phospho-ERK1/2 (1:1000, 4370, CST), rabbit anti-ERK1/2 (1:1000, 4695, CST) and mouse anti‐GAPDH (1:3000, TA-08, ZSGB-BIO) for immunoblotting. The ECL detection system (EMD Millipore) was used to assess protein expression. GAPDH was used as the internal control. The experiments were conducted in triplicate.

### Cell proliferation assay

A CellTiter-Glo luminescent cell viability assay kit (Promega Corporation) was used to measure cell proliferation on an INFINITE 200 Pro multimode reader (TECAN) according to the manufacturer’s instructions. Prior to transfection, 5×10^3^ BC cells were placed into 96-well plates (Corning) in final volumes of 120 µl, and four 96-well plates were incubated for 0, 24, 48 and 72 h. The CellTiter-Glo assays provide a homogeneous method for determining the number of viable cells in culture based on quantitation of ATP, which indicates the presence of metabolically active cells at different times according to the manufacturer’s protocol. The experiments were conducted in triplicate.

### Transwell assay

The cell migration assay was evaluated using Transwell chambers (8 μm, Corning). A total of 3×10^4^ cells was suspended in serum-free medium and placed into the upper chamber of each insert, and 600 µl of complete medium was added to the bottom well. After incubation, the cells that did not migrate were removed with cotton swabs. The metastatic cells on the bottom surface of the membrane were fixed with 4% paraformaldehyde solution and stained with 0.1% crystal violet. Images of three random fields were captured from each membrane, and the number of migratory cells was counted. The experiments were conducted in triplicate.

### Wound healing assay

Before the second transfection a scratch was created using a sterile 200 μl pipettor tip in 12-well plates. Cells were then transfected at 48 h and monitored at 72 h-96 h, and images of three random fields were captured from each well. The percentage of wound healing was recorded. The experiments were conducted in triplicate.

### Target gene screening of miRNA

First, to identify the potential target gene and biological function of miR-361-3p, we analyzed the differentially expressed genes (DEGs) by RNA sequencing (RNA-seq) between the total RNA of miR-361-3p transfectants and miR-NC-transfected MDA-MB-231 cells. The selection criteria for candidate target genes among downregulated genes are *P* < 0.05 and log_2_FC < −1. To further identify the putative target of miR-361-3p, online miRNA target analysis algorithms (miRWalk database and TargetScan 7.2) were used, and the top genes from the above datasets were further analyzed through the results of previous studies.

### Gene ontology enrichment analysis

To investigate the potential molecular biological functions of miR-361-3p, we employed the online bioinformatics tool DAVID (https://david.ncifcrf.gov/) for Gene Ontology (GO) enrichment analysis. This analysis focused on DEGs between BC cells with overexpression of miR-361-3p and their controls. Fisher’s exact test and the *χ*^*2*^*-test* were executed to select the significant GO terms. The threshold of significance was determined by a *P* < 0.05.

### Gene set enrichment analysis

We utilized the GSEA 4.1.0 software to conduct Gene Set Enrichment Analysis (GSEA) for investigating the differences in biological pathways between BC samples with high and low expression of *PAI-1* in The Cancer Genome Atlas (TCGA) dataset (provisional dataset, 2023). This analysis included samples with complete follow-up information and gene expression profiling data (n = 1,009). The *P* value and normalized enrichment score (NES) were used to sort the enriched pathways, with 1,000 sorts per analysis.

### Luciferase reporter assay

According to the putative binding sites predicted for *miR-361-3p* with the full-length 3’-untranslated region (3’-UTR) fragment of *ETV7* and *BATF2* by TargetScan 7.2 (http://www.targetscan.org), the wild-type or mutant fragment of the *ETV7* 3'-UTR and *BATF2* 3'-UTR containing the predicted *miR-361-3p*-binding site were cloned into the pmirGLO Dual-Luciferase miRNA Target expression reporter vector (Promega Corporation). MDA-MB-231 cells were cotransfected with 200 nM miRNA and 0.4 μg pmirGLO vector. The cells were transfected using Lipofectamine 2000 for 24 h and analyzed using the Dual-Glo Luciferase Assay System (Promega Corporation). The relative luciferase activity was calculated by normalizing the luminescence intensity of firefly luciferase reactivity to that of Renilla luciferase reactivity, according to the manufacturer’s protocol. The experiments were conducted in triplicate.

### Xenografted tumour models

This animal experiment was approved by the Experimental Animal Welfare and Ethics Review Committee of the Institute of Zoology, Chinese Academy of Sciences (Approval Number: #IOZ-IACUC-2023–083). Six-week-old female BALB/c nude mice (n = 14) were purchased from Spfbiotech, China. T47D cells (1 × 10^7^ cells in 120ul PBS) were injected into the fourth mammary fat pad of the mice. As shown in Fig. 5A, 3 days post-injection, the mice were randomly divided into two equal groups. Each group received tail vein injections of either EXO-miR-NC or EXO-miR-361-3p particles, with a dosage of 4 × 10^11^ particles per mice, administered every 3 days for a total of three injections. Tumor sizes were measured at 3-day intervals, and tumor volumes were calculated using the formula: volume = (longest diameter) × (shortest diameter)^2^ × 0.5. After 15 days, the mice were euthanized, the blood was collected for analysis of plasma exosomal miRNA. Additionally, the excised tumors from the mice were weighed and then subjected to various analyses, including hematoxylin and eosin (H&E) staining, qRT‒PCR, and WB assays.

### Statistical analysis

SPSS version 25 (IBM Corp, Armonk, N.K. USA) and R software version 4.0.5 (R core Team, Vienna, Austria) were used to statistically analyze the data. GraphPad Prism 7 (GraphPad Software Inc., San Diego, CA, USA) was used for analysis and image drawing. For categorical variables, a chi-square test or Fisher's exact probability test was used. For continuous variables, values of *t-test*, *ANOVA test* or *Mann–Whitney U-test* was used.* P* < 0.05 were considered statistically significant. Linear regression models were deployed to detect the linear trend test. Each experiment was repeated at least three times.

## Results

### Plasma exosomal miR-361-3p levels gradually increase with malignant progression in BC patients

**Fig. 1:**
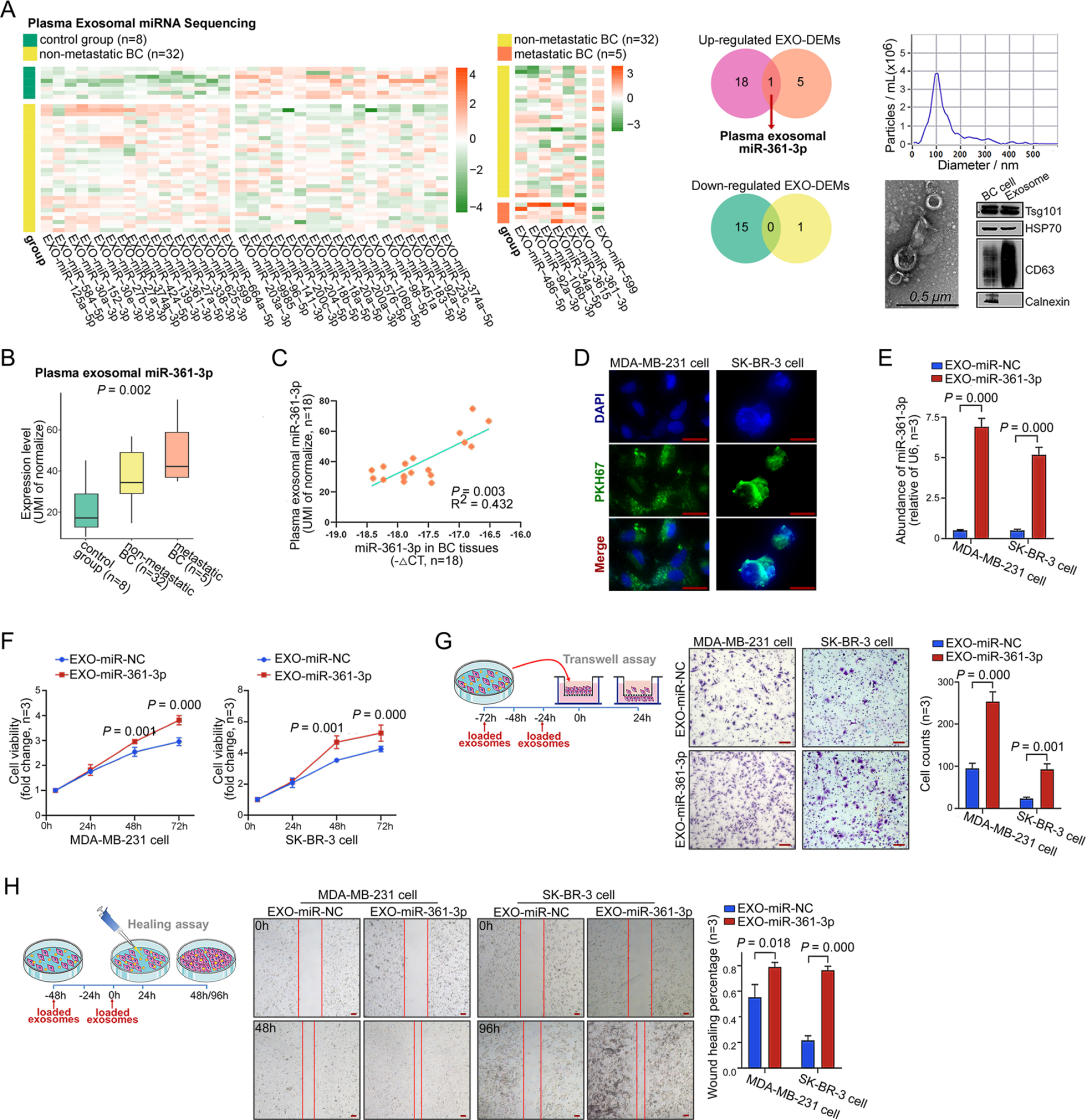
Plasma exosomal miR-361-3p is associated with malignant progression in BC patients. **A** Heatmaps demonstrate plasma exosomal DEMs between the control group and nonmetastatic BC patients, as well as between nonmetastatic and metastatic BC (TPM ≥ 30, *P* < 0.05, |log_2_FC|> 0.5); Venn diagrams point out plasma exosomal miRNAs that were consistently changed; and the three images on the right side show the identification of plasma exosomes. **B** Box line plot showing elevated expression of plasma exosomal miR-361-3p with BC malignant progression. **C** Plasma exosomal miR-361-3p was positively correlated with the miR-361-3p expression level in BC tissues. **D** Green particles are PKH67-labeled exosomes entering BC cells, and blue are DAPI-labeled BC cell nuclei. Scale bars, 20 μm. **E** qRT‒PCR was performed to detect the abundance of miR-361-3p in BC cells after coculture for 72 h with exosomes. **F** The effect of EXO-miR-361-3p on BC cell proliferation was tested using the CellTiter-Glo kit. **G**–**H** Transwell and scratch healing assays were used to test the effect of EXO-miR-361-3p on BC cell migration, and the schematic demonstrates the time intervals of exosome loading. Scale bars, 100 μm

First, we isolated and identified plasma exosomes before miRNA-seq: (1) NTA analysis showed that the isolated vesicle particles were mainly 100 nm in diameter, which is consistent with the diameter of exosome particles; (2) TEM analysis provided evidence of the morphological integrity of exosomes; and (3) WB analysis of TSG101, HSP70 and CD63 protein expression proved that the isolated products were exosomes, while the absence of calnexin protein expression ruled out cellular contamination. Next, differential expression miRNAs (DEMs) analysis showed that 19 miRNAs were upregulated and 15 miRNAs were downregulated in plasma exosomes from nonmetastatic BC patients compared to controls. Six miRNAs were upregulated and one miRNA was downregulated in metastatic BC patients compared to nonmetastatic BC patients. Notably, only miR-361-3p was upregulated in both groups (Fig. 1A). As shown in Fig. 1B, plasma exosomal miR-361-3p gradually increased with malignant progression in BC patients. Subsequently, the qRT‒PCR results showed that the expression levels of miR-361-3p were positively correlated between plasma exosomes and tumor tissues in BC patients. (Fig. 1C). Further comparing plasma exosomal miR-361-3p expression levels and patient clinical factors, we found that elevated plasma exosomal miR-361-3p is closely related to higher grade (*P* = 0.046), larger tumor size (*P* = 0.030), and advanced stage (*P* = 0.039) (Additional file 1: Figure S1A). The above findings suggest that plasma exosomal miR-361-3p may be associated with malignant progression in BC patients.

### Exosomal miR-361-3p promotes BC cell migration and proliferation

To evaluate the influence of exosomal miR-361-3p on BC cells, we cocultured recipient BC cells (MDA-MB-231 and SK-BR-3) with either EXO-miR-361-3p or EXO-miR-NC. After 12 h, PKH67-labeled vesicles were observed within the cytoplasm of MDA-MB-231 and SK-BR-3 cells (Fig. 1D). Furthermore, after 72 h, qRT‒PCR analysis showed that the relative abundance of miR-361-3p was significantly higher in cells exposed to EXO-miR-361-3p than in those treated with EXO-miR-NC (Fig. 1E). Finally, CellTiter-Glo assays demonstrated a notable growth increase in BC cells cocultured with EXO-miR-361-3p, particularly at 72 h (Fig. 1F). Transwell and scratch wound healing assays indicated enhanced migratory and wound healing capabilities in BC cells cocultured with EXO-miR-361-3p (Fig. 1G, H). These findings highlight that exosome-delivered miR-361-3p could enter BC cells and enhance their tumor-promoting behaviors.

To more comprehensively elucidate the role of miR-361-3p in BC cells, we performed miR-361-3p knockdown experiments in MDA-MB-231 cell line. The results indicated that BC cells transfected with the miR-361-3p inhibitor exhibited a significant reduction in miR-361-3p expression levels compared to control cells (Additional file 1: Figure S2A). Furthermore, these cells demonstrated markedly decreased capabilities in cell proliferation, wound healing, and migration (Additional file 1: Figure S2B-D).

### *ETV7* and *BATF2* are two novel targets of miR-361-3p in BC cells

**Fig. 2:**
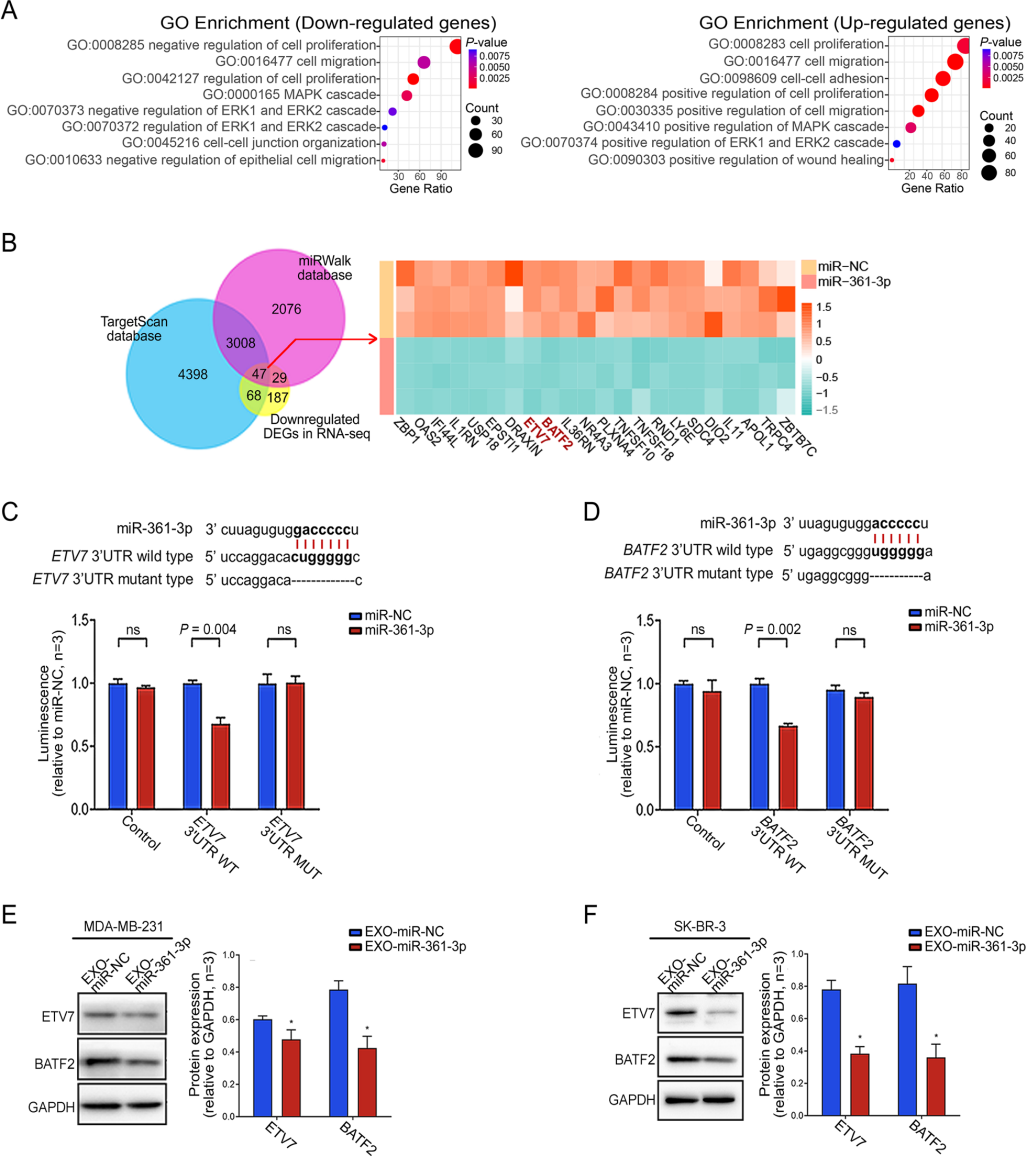
*ETV7* and *BATF2* are two new targets of miR-361-3p. **A** The GO analysis revealed distinct pathways enriched by genes that are either downregulated or upregulated in BC cells overexpressing miR-361-3p. **B** Intersection of our RNA-seq results (displayed as a heatmap) and miRNA target prediction algorithms from 2 databases. **C**–**D** miR-361-3p putative targeting sites in the wild-type and mutant ETV7 3'UTR and BATF2 3'UTR, and luciferase activity assays were performed to confirm the direct binding efficiency of miR-361-3p and its targets ETV7 and BATF2. **E**–**F** Relative protein expression of ETV7 and BATF2 after coculturing BC cells with EXO-miR-361-3p/EXO-miR-NC. (*P < 0.05)

To investigate the biological role of miR-361-3p in BC cells and to identify its target molecules, we conducted RNA-seq analysis. First, the results of GO analysis showed that downregulated genes in cells overexpressing miR-361-3p were mainly enriched in pathways associated with inhibiting tumor proliferation and migration. In contrast, upregulated genes were mainly enriched in pathways associated with promoting tumor proliferation and migration (Fig. 2A). These findings supported that miR-361-3p plays a key role in enhancing the viability of BC cells.

Furthermore, among 331 candidate target genes, 47 overlapped with predicted target genes from an online database (Fig. 2B). Upon reviewing the available studies on the 47 DEGs, we found that *ETV7* and *BATF2* are considered repressor genes in some cancers and can be involved in suppressing malignant progression in patients [21–24]. However, their specific roles in BC patients are unknown. Based on this, we focused on these two potential target genes.

We initially predicted miR-361-3p binding sites in the 3'-UTR of *ETV7* and *BATF2* mRNA using TargetScan 7.2. Luciferase reporter gene assays showed that miR-361-3p overexpression significantly inhibited luciferase activity relative to that in the miR-NC group (Fig. 2C, D). Notably, these effects were abolished upon mutation of the predicted binding site. This suggests that miR-361-3p directly downregulates *ETV7* and *BATF2* by binding to their 3'UTRs. Subsequent WB experiments in MDA-MB-231 and SK-BR-3 cell lines confirmed that compared with those in the control group, the protein expression levels of ETV7 and BATF2 were reduced after 72 h of coculture with EXO-miR-361-3p (Fig. 2E, F). Finally, we further examined the expression levels of ETV7 and BATF2 in cells where the expression level of miR-361-3p was downregulated. The results showed that the knockdown of miR-361-3p led to an increase in the expression levels of ETV7 and BATF2 in BC cells (Additional file 1: Figure S2E). The above results supported that *ETV7* and *BATF2* are two novel targets of miR-361-3p in BC cells.

### Knockdown of *ETV7* and *BATF2* increases *PAI-1* expression and promotes the migration and proliferation of BC cells

**Fig. 3:**
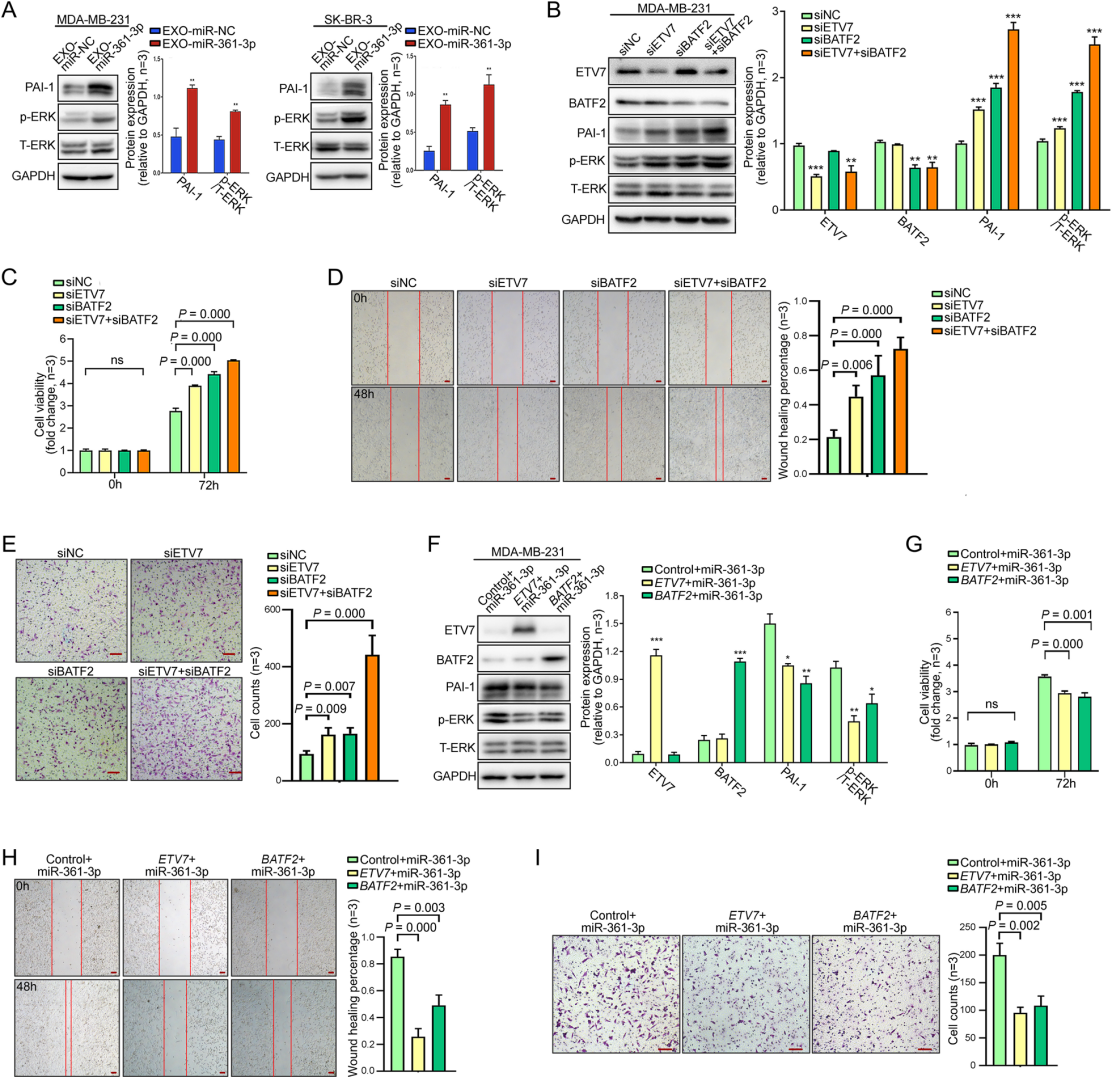
ETV7 and BATF2 negatively regulate the PAI-1/ERK pathway as well as the proliferation and migration of BC cells. **A** Relative protein expression of PAI-1, p-ERK and T-ERK after coculturing BC cells with EXO-miR-361-3p/EXO-miR-NC. **B**, **F** Relative expression of ETV7, BATF2, PAI-1 and p-ERK/T-ERK protein after ETV7 and/or BATF2 knockdown or overexpression (*P < 0.05, **P < 0.01, ***P < 0.001). CellTiter-Glo assays (**C**, **G**), wound healing assays (**D**, **H**) and Transwell assays (**E**, **I**) were performed to test the effect of ETV7 and/or BATF2 knockdown or overexpression on BC cell proliferation and metastasis. Scale bars, 100 μm

Further analyses of pathways related to cellular functions revealed that both *ETV7* and *BATF2* were considered transcriptional repressors of a common downstream gene, *PAI-1*, in previous studies [24–26]. Plasminogen activator inhibitor 1 (PAI-1), also known as Serpine1, is a serine protease inhibitor and the most prominent negative regulator of the proteolytic urokinase plasminogen activator system. Previous studies showed that upregulated PAI-1 could activate the extracellular signal-regulated kinase (ERK) signaling pathway and increase the phosphorylation level of ERK, which promotes the proliferation and migration of BC cells [27–33]. Consistent with this context, we observed increased PAI-1 and phosphorylated ERK levels after 72 h of coculture of BC cells with EXO-miR-361-3p (Fig. 3A).

Subsequently, siETV7 and/or siBATF2 were transfected into MDA-MB-231 cells, and the results were confirmed by WB (Fig. 3B). ETV7 or/and BATF2 knockdown increased PAI-1 expression and activated ERK, particularly in cotransfected BC cells. Transwell, wound healing, and CellTiter-Glo assays demonstrated enhanced migration and proliferation in ETV7- or BATF2-knockdown BC cells. Notably, cotransfection with siETV7 and siBATF2 exerted a stronger effect (Fig. 3C–E). Our results confirmed that ETV7 and/or BATF2 knockdown elevates the PAI-1/ERK pathway, increasing BC cell tumor-promoting behaviors.

### Overexpression of ETV7 or BATF2 rescues miR-361-3p-induced enhancement of BC cell viability

To further verified the regulatory relationship between miR-361-3p and *ETV7* or *BATF2*, we cotransfected MDA-MB-231 cells with either the *ETV7* plasmid, the *BATF2* plasmid, or control plasmid, along with miR-361-3p. The results indicated that upregulation of ETV7 and BATF2 could rescue the elevated expression levels of PAI-1 and p-ERK caused by miR-361-3p overexpression (Fig. 3F). Furthermore, this upregulation also rescued the enhanced cell viability induced by miR-361-3p overexpression (Fig. 3G–I).

### MiR-361-3p promotes proliferation and migration by upregulating the PAI-1/ERK pathway in BC cells

**Fig. 4:**
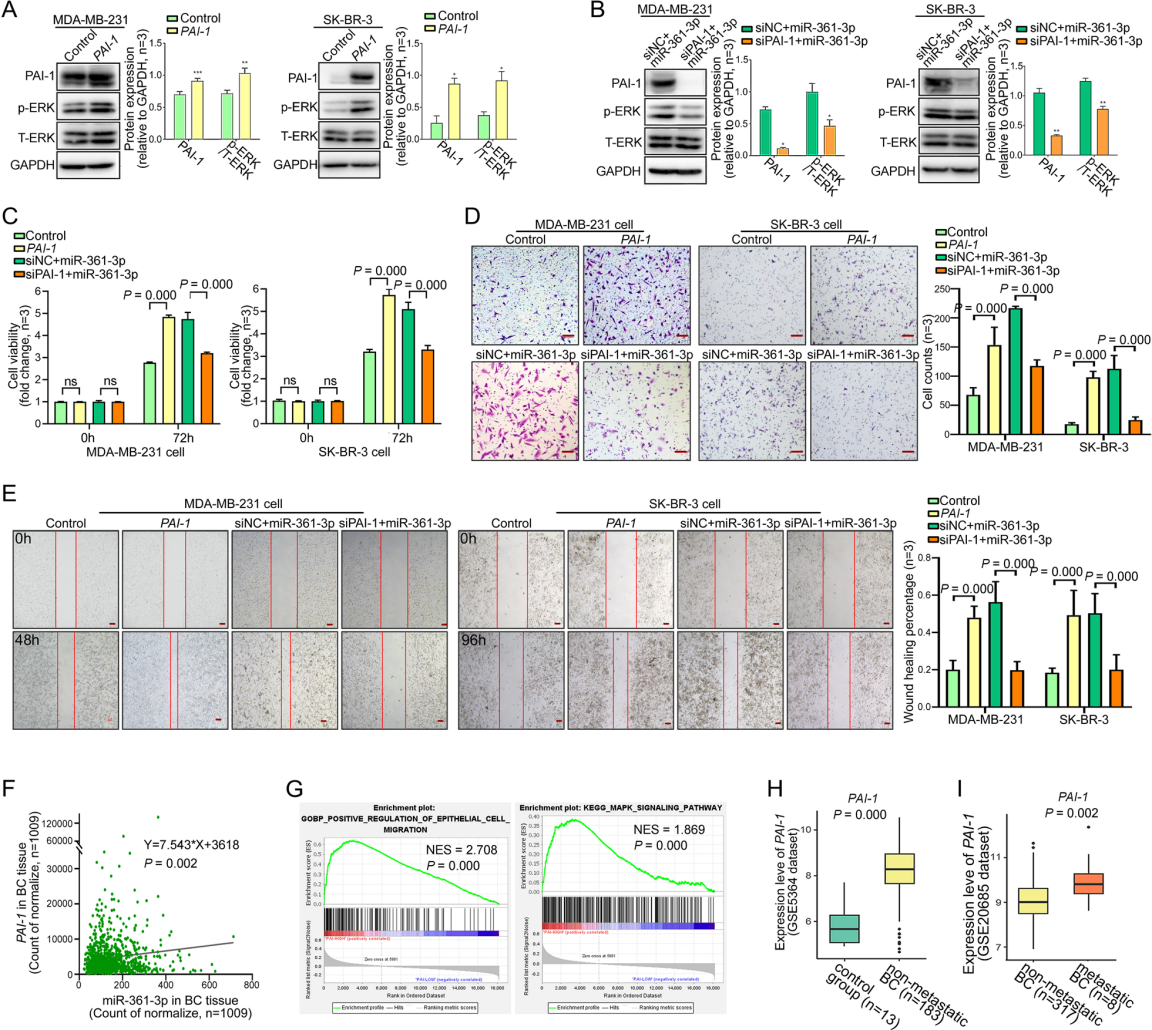
MiR-361-3p promotes proliferation and migration by upregulating the PAI-1/ERK pathway in BC cells. **A**, **B** Relative expression of PAI-1 and p-ERK/T-ERK protein after transfection with PAI-1 plasmid alone or in combination with miR-361-3p and siPAI-1. CellTiter-Glo assays (**C**), Transwell assays (**D**), and wound healing assays (**E**) were performed to test the effect of overexpression of PAI-1 alone or in combination with miR-361-3p and knockdown of PAI-1 on BC cell proliferation and migration. Scale bars, 100 μm. **F** Analyzing the correlation between miR-361-3p and *PAI-1* expression level using TCGA dataset. **G** GSEA analysis based on *PAI-1* expression level using TCGA dataset. **H**, **I** Expression trend of *PAI-1* in normal breast tissue, nonmetastatic BC tissue, and metastatic BC tissue. (*P < 0.05, **P < 0.01, ***P < 0.001)

We transfected the *PAI-1* plasmid and control plasmid into MDA-MB-231 and SK-BR-3 cell lines, and cotransfected siPAI-1 and miR-361-3p or siNC and miR-361-3p into these cell lines (Fig. 4A–E). The results showed that overexpression of PAI-1 or miR-361-3p increased activated ERK, while knockdown of PAI-1 expression counteracted ERK activation in miR-361-3p-overexpressing BC cells. Transwell, wound healing and CellTiter-Glo assays showed that PAI-1 or miR-361-3p overexpression significantly promoted the migration and proliferation of BC cells, while knockdown of PAI-1 abolished the promoting effect of miR-361-3p on cell proliferation and metastasis in BC cells.

Next, we utilized the TCGA dataset to conduct a further analysis of the relationship and function of the miR-361-3p/PAI-1/ERK axis. The results showed a positive correlation between the expression levels of miR-361-3p and *PAI-1* in BC tissues, suggesting a positive relationship between these genes (Fig. 4F). Moreover, the GSEA results indicated that high expression levels of *PAI-1* are significantly associated with the promotion of epithelial cell migration and the activation of the MAPK signaling pathway (Fig. 4G). Finally, analyses of the GEO database revealed that *PAI-1* expression was significantly elevated in nonmetastatic BC tissues compared to normal breast tissues in the GSE5364 dataset (n = 196) and in metastatic BC tissues compared to nonmetastatic BC tissues in the GSE20685 dataset (n = 325) (Fig. 4H, I). Such results suggest that the expression level of *PAI-1* gradually increases with the degree of BC malignancy, which is consistent with the expression trend of miR-361-3p in plasma exosomes. The above results further support that exosomal miR-361-3p can enhance the migration and proliferation of BC cells by activating the PAI-1/ERK pathway, ultimately promoting the malignant progression of BC.

### Elevated plasma exosomal miR-361-3p promotes the progression of BC in xenograft tumor mice

**Fig. 5:**
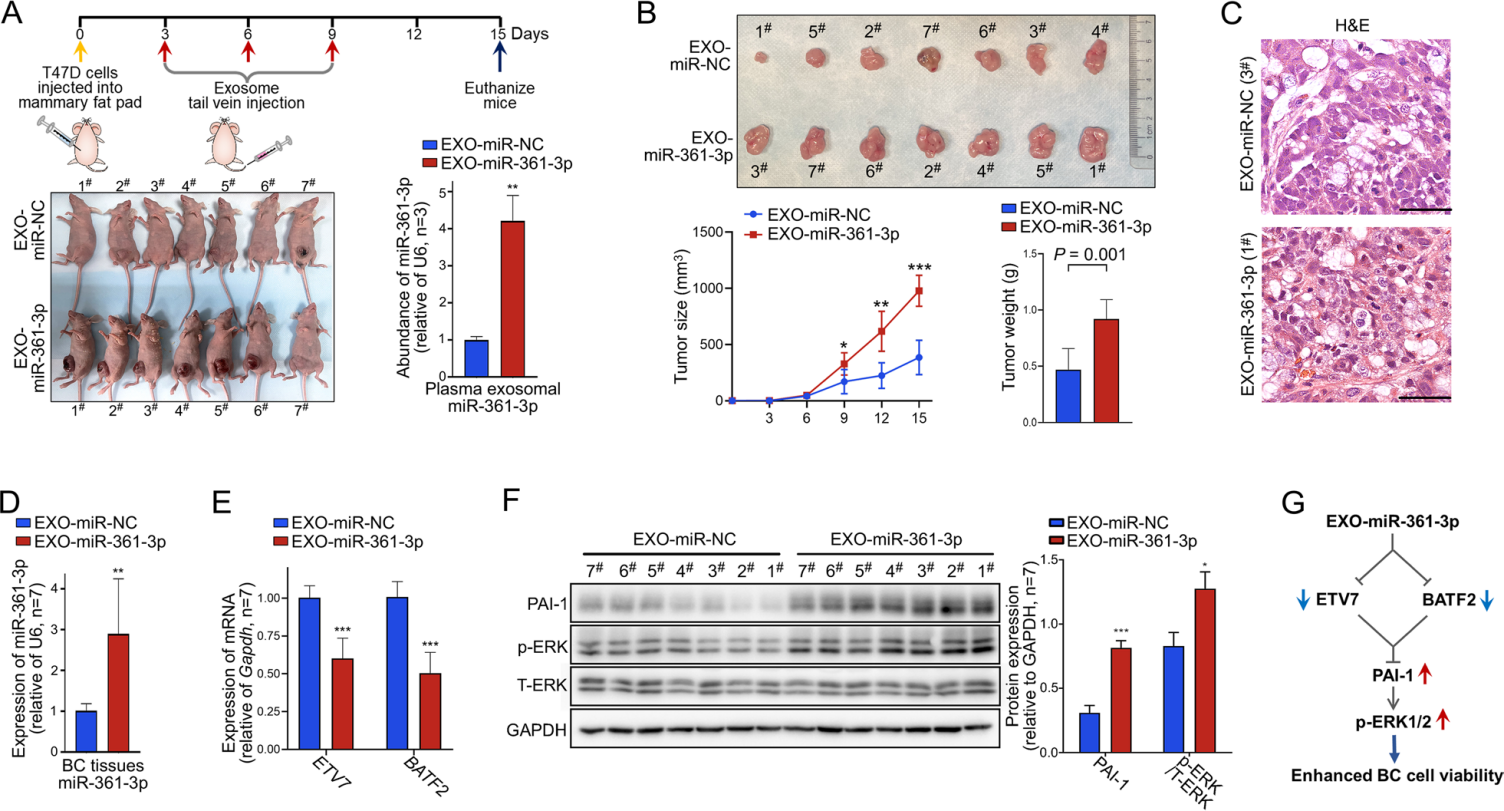
Elevated plasma exosomal miR-361-3p promotes the malignant progression of BC in mice. **A** *Upper panel*: A schematic diagram of the animal experiments. *Lower panel*: A T47D cell xenograft model in female BALB/c nude mice was established. Mice exhibiting a high abundance of plasma exosomal miR-361-3p, as well as control mice, were obtained through the tail vein injection of EXO-miR-361-3p or EXO-miR-NC, respectively. **B** Differences in volume, weight and growth rate of murine tumors in the EXO-miR-361-3p group compared to the control group. **C** H&E staining of murine tumor tissues (×40). **D**–**F** Compare the expression levels of miR-361-3p, *ETV7*, *BATF2*, and the PAI-1/ERK pathway in excised tumors of the two groups of mice. **G** Proposed model of exosomal miR-361-3p targeting *ETVT* and *BATF2* to upregulate the PAI-1/ERK pathway, leading to increased viability in BC cells. (*P < 0.05, **P < 0.01, ***P < 0.001)

We further validated the effect of exosomal miR-361-3p on BC progression using murine xenograft models. Mice were firstly injected with T47D cells in mammary fat pad, followed by exosomal particles through the tail vein. The results indicated that mice injected with EXO-miR-361-3p shown a significantly higher abundance of miR-361-3p in their plasma exosomes compared to those injected with EXO-miR-NC (Fig. 5A). Furthermore, both the volume and weight of the breast tumors in the EXO-miR-361-3p group were significantly greater than those in the EXO-miR-NC group (Fig. 5B, C).

Furthermore, we noted that mice injected with EXO-miR-361-3p demonstrated a significant increase in miR-361-3p expression levels and a marked decrease in *ETV7* and *BATF2* expression in tissues, relative to the EXO-miR-NC group (Fig. 5D, E). Additionally, WB experiments indicated that the expression levels of PAI-1 and p-ERK were significantly upregulated (Fig. 5F). These in vivo results are consistent with the in vitro results, further suggesting that exosomal miR-361-3p can activate the PAI-1/ERK pathway by targeting *ETV7* and *BATF2*, thereby enhancing cell viability and ultimately leading to malignant progression of BC (Fig. 5G).

## Discussion

In this study, we have discovered for the first time that elevated plasma exosomal miR-361-3p of BC patients is positively correlated with disease progression. Our further in vivo and in vitro experimental results suggest that plasma exosomal miR-361-3p could serve as a potential marker for assessing the disease progression in BC patients.

Previous studies have shown that abnormally expressed plasma exosomal miR-361-3p is associated with the onset and progression of some diseases. For instance, a study on colorectal cancer demonstrated a significant correlation between elevated plasma exosomal miR-361-3p and poor prognosis in colorectal cancer patients, proposing its potential as a prognostic biomarker. The study further revealed that hypoxic colorectal cancer cells secrete large amounts of exosomal miR-361-3p, which enhances the proliferation and inhibits apoptosis in adjacent colorectal cancer cells, thereby contributing to the malignant progression of the cancer [34]. Additionally, another research found a notable association between increased plasma exosomal miR-361-3p and bicuspid aortic valve disease [35]. However, no previous research had established a connection between abnormal expression of plasma exosomal miR-361-3p and the progression of BC in patients. Our study is the first to reveal this association. We found that plasma exosomal miR-361-3p gradually increases with the progression of BC by analyzing plasma exosomal miRNA profiles from healthy controls, as well as non-metastatic and metastatic BC patients. Supporting this, our cellular experiments demonstrated that exosome-delivered miR-361-3p enhances the vitality of BC cells. Moreover, in a murine xenograft tumor model, we further confirmed that elevated levels of plasma exosomal miR-361-3p can accelerate BC progression in mice. Consequently, we propose that plasma exosomal miR-361-3p has the potential to be a marker for monitoring disease progression in BC patients.

Previous research outcomes have demonstrated that miR-361-3p plays a crucial regulatory role in various cancer types. In some cancers, miR-361-3p acts as an oncogene. For instance, Jisheng et al. found that in pancreatic cancer, miR-361-3p can activate the ERK pathway by targeting *DUSP2*, inducing EMT in pancreatic cancer cells and ultimately promoting metastasis [36]. Jie Li et al. discovered that in colon cancer, exosomal miR-361-3p produced by hypoxic cells can enter colon cancer cells, target TNF receptor-associated factor 3 to inhibit apoptosis and promote cell growth [34]. Our previous research found that miR-361-3p in BC cells can regulate the E2F1/P73 pathway to inhibit apoptosis [37]. In this study, we further identified novel target genes for miR-361-3p, *ETV7* and *BATF2*, and demonstrated through in vivo and in vitro experiments that exosomal miR-361-3p can activate the PAI-1/ERK pathway by inhibiting ETV7 and BATF2, thereby enhancing the vitality of BC cells. Contrastingly, some studies have shown that miR-361-3p can act as a tumor suppressor gene, such as in gastric cancer, where it targets *TGFB1* or *RABL6* to inhibit the progression and metastasis of gastric cancer [38, 39]. These differing results could be attributed to the complex roles of miR-361-3p in tumor growth and progression, which likely vary depending on the specific cellular context and the stages of cancer development. However, these findings emphasize the significant regulatory role of miR-361-3p in tumor cells and underscore its potentially extensive influence on tumor initiation and progression.

Our study has some limitations. While our results indicate that plasma exosomal miR-361-3p is expressed at higher levels in patients with metastatic BC, implying its potential as a marker for distant metastasis, the sample size for this subgroup is currently small. To address this issue, we will focus on collecting plasma samples from patients with metastatic BC in order to more fully validate our findings.

This study uncovers a novel regulatory mechanism of exosomal miR-361-3p in enhancing the vitality of BC cells. It offers valuable information for evaluating disease progression and identifying potential intervention targets in BC patients.

## Conclusions

Our findings demonstrate that elevated plasma exosomal miR-361-3p expression is associated with malignant progression in BC patients. Exosome-delivered miR-361-3p can activate the PAI-1/ERK pathway by targeting *ETV7* and *BATF2*, thereby enhancing the viability of BC cells. These outcomes suggest that elevated plasma exosomal miR-361-3p could potentially serve as a biomarker for malignant progression in BC patients.

### Supplementary Information

**Additional file 1: Figure S1.** The correlation between the expression level of plasma exosomal miR-361-3p and clinical factors in BC patients. (A) The box plot shows the relationship between the plasma exosomal miR-361-3p and the clinical factors of BC patients. **Figure S2.** Downregulation of miR-361-3p expression inhibits BC cell viability (A) qRT‒PCR was performed to detect the expression level of miR-361-3p in BC cells transfected with miR-361-3p inhibitor or miR-NC inhibitor. CellTiter-Glo assays (B), wound healing assays (C) and Transwell assays (D) were performed to test the effect of miR-361-3p knockdown on BC cell proliferation and metastasis. Scale bars, 100 μm. (E) Relative protein expression of ETV7 and BATF2 after transfecting with miR-361-3p inhibitor or miR-NC inhibitor. (*P < 0.05, **P < 0.01).

## Data Availability

Data available on request from the authors.

## Abbreviations

BC: Breast cancer
miRNA: MicroRNA
miR-seq: MiRNA sequencing
UMIs: Unique molecular identifiers
RNA-seq: RNA sequencing
TPM: Transcripts per million
DEMs: Differentially expressed miRNAs
DEGs: Differentially expressed genes
NC: Negative control
GEO: Gene expression omnibus
NTA: Anoparticle tracking analysis
TEM: Transmission electron microscopy
WB: Western blotting
qRT‒PCR: Quantitative real-time polymerase chain reaction
3’-UTR: 3’-Untranslated region
FC: Fold change
GO: Gene ontology
GSEA: Gene set enrichment analysis
TCGA: The Cancer Genome Atlas
NES: Normalized enrichment score
H&E: Hematoxylin and eosin
