# Supplementary material for: Exosomal miR-361-3p promotes the viability of breast cancer cells by targeting ETV7 and BATF2 to upregulate the PAI-1/ERK pathway
Source: J Transl Med. 2024 Jan 28;22:112. doi: 10.1186/s12967-024-04914-4 (PMC10823750; doi:10.1186/s12967-024-04914-4)
Supplement: Supplementary file 1 — Additional file 1: Figure S1. The correlation between the expression level of plasma exosomal miR-361-3p and clinical factors in BC patients. (A) The box plot shows the relationship between the plasma exosomal miR-361-3p and the clinical factors of BC patients. Figure S2. Downregulation of miR-361-3p expression inhibits BC cell viability (A) qRT‒PCR was performed to detect the expression level of miR-361-3p in BC cells transfected with miR-361-3p inhibitor or miR-NC inhibitor. CellTiter-Glo assays (B), wound healing assays (C) and Transwell assays (D) were performed to test the effect of miR-361-3p knockdown on BC cell proliferation and metastasis. Scale bars, 100 μm. (E) Relative protein expression of ETV7 and BATF2 after transfecting with miR-361-3p inhibitor or miR-NC inhibitor. (*P < 0.05, **P < 0.01). [file 12967_2024_4914_MOESM1_ESM.docx]

**
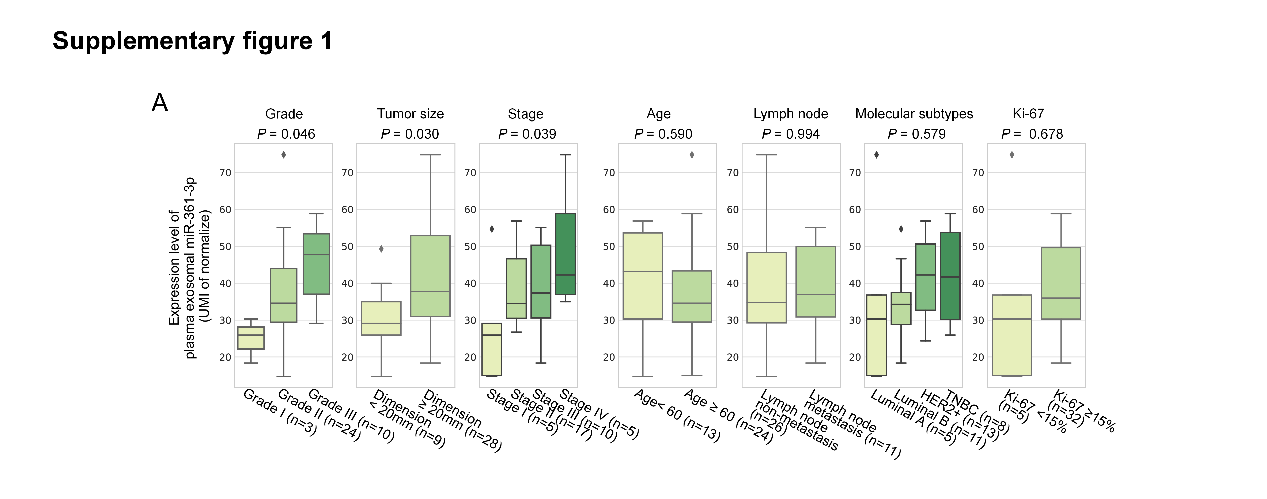
**

**Additional Figure 1.** (A) The box plot shows the relationship between the plasma exosomal miR-361-3p and the clinical factors of BC patients.

**
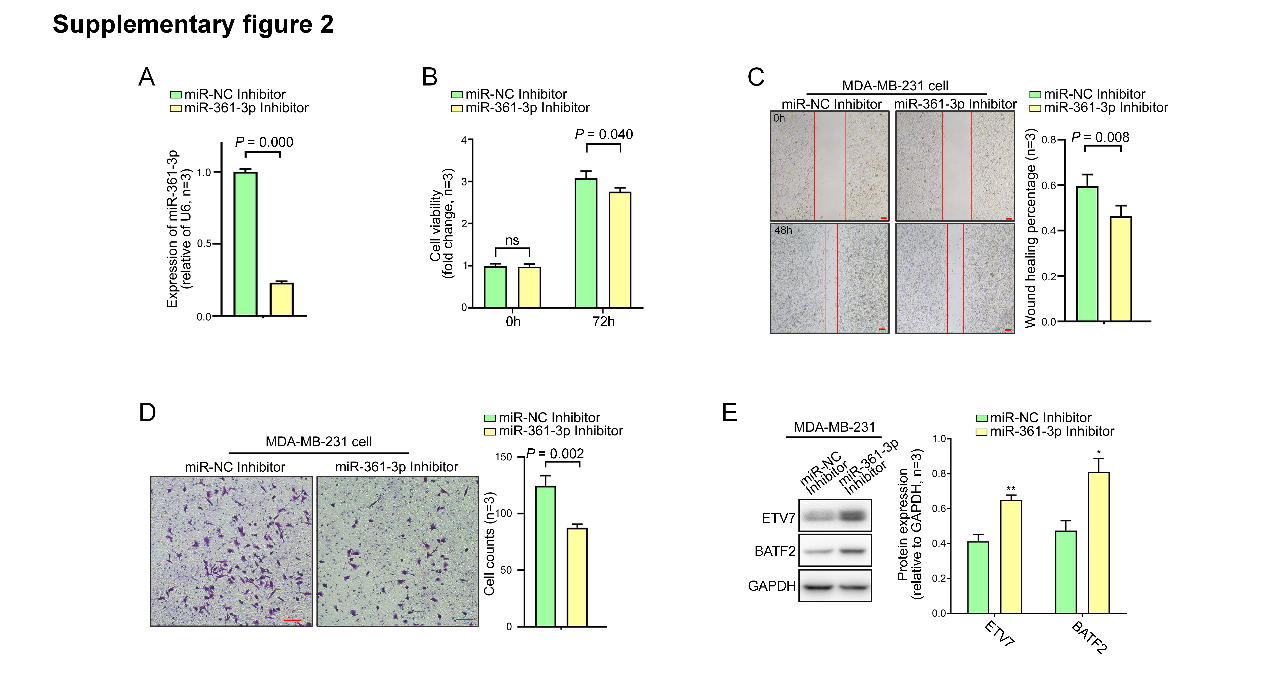
**

**Additional Figure 2. Downregulation of miR-361-3p expression inhibits BC cell viability** (A) qRT‒PCR was performed to detect the expression level of miR-361-3p in BC cells transfected with miR-361-3p inhibitor or miR-NC inhibitor. CellTiter-Glo assays (B), wound healing assays (C) and Transwell assays (D) were performed to test the effect of miR-361-3p knockdown on BC cell proliferation and metastasis. Scale bars, 100 μm. (E) Relative protein expression of ETV7 and BATF2 after transfecting with miR-361-3p inhibitor or miR-NC inhibitor. (*P <0.05, ***P* < 0.01).
